# Intrinsic capacity of older people in the community using WHO Integrated Care for Older People (ICOPE) framework: a cross-sectional study

**DOI:** 10.1186/s12877-022-02980-1

**Published:** 2022-04-08

**Authors:** Angela Y. M. Leung, Jing Jing Su, Elsa S. H. Lee, Jeff T. S. Fung, Alex Molassiotis

**Affiliations:** 1 World Health Organization Collaborating Centre (WHOCC) for Community Health Services, School of Nursing, The Hong Kong Polytechnic University, Hong Kong SAR, China; 2 Centre for Gerontological Nursing, School of Nursing, The Hong Kong Polytechnic University, Hong Kong SAR, China; 3 School of Nursing, The Hong Kong Polytechnic University, Hong Kong SAR, China; 4 Hong Kong Sheng Kung Hui Welfare Council Ltd., Hong Kong SAR, China

**Keywords:** ICOPE, Intrinsic capacity, Older adults, Healthy aging, Integrated care

## Abstract

**Background:**

The World Health Organization (WHO) published the Integrated Care for Older People (ICOPE) framework to guide assessing and promoting intrinsic capacity of older adults. This study, adopting the WHO ICOPE framework, assessed the intrinsic capacity impairment and investigated the relationship among intrinsic capacity, social engagement, and self-care capacity on performing activities of daily living. It also assessed the sensitivity of the initial brief screening and the detailed full assessment.

**Methods:**

This is a cross-sectional study conducted in 11 community centers in Hong Kong. Intrinsic capacity was assessed in two steps identical to WHO ICOPE handbook: using WHO ICOPE brief screening tool (step 1) and detailed full assessment (step 2) to assess the intrinsic capacity domains of locomotion, cognition, vitality, psychological well-being, and sensory capacity (hearing and vision). Structural equational modeling analysis was used to examine the relationship among intrinsic capacity, social engagement, and self-care capacity, and the mediating role of intrinsic capacity in the relationships.

**Results:**

A total of 304 older adults with a mean age 76.73 (SD = 7.25) years participated in WHO ICOPE Step 1 brief screening, and 221 participants (72.7%) showed intrinsic capacity impairment. After completing Step 2 full assessment, 202 participants (66.4%) had one or more impaired intrinsic capacity domains. The overall sensitivity and specificity of the screening tool were 95% and 57.6% respectively, whereas the sensitivity of each domain ranged from 74.7% to 100%. The percentage of impairment in locomotion (117, 39.8%), cognition (75, 25.5%), psychological well-being (34, 11.6%), vision (75, 24.7%), hearing capacity (82, 27.9%), and vitality (8, 2.7%). People in younger old age (β = -0.29, *p* < 0.001), with more education (β = 0.26, *p* < 0.001), and absence of hypertension (β = -0.11, *p* < 0.05) were more likely to have better intrinsic capacity. Intrinsic capacity was positively associated with self-care capacity in performing activities of daily living (β = 0.21, *p* < 0.001) and social engagement (β = 0.31, *p* < 0.001).

**Conclusions:**

The ICOPE screening tool is a sensitive instrument to detect intrinsic capacity impairment among community-dwelling older adults and it does not demand substantial workforce; its use is worthy to be supported. The intrinsic capacity impairment in community-dwelling older adults are prevalent, in particular, in locomotor and cognitive capacity. Actions should be taken to slow or prevent the impairment, support self-care and social engagement in old age.

## Background

The world population is aging rapidly and the population of older adults is projected to rise from one billion to 2.1 billion from 2019 to 2050 [1]. The worldwide average healthy life years at the age of 60 years ranged widely between 9.79 in Lesotho to 20.39 in Japan, implying distinctively diversified health conditions for older adults [2]. Thus, the World Health Organization (WHO) has released the World Report on Ageing and Health to reframe and re-orientate global action for healthy aging [3].

The World Report took a capacity-based approach to frame healthy aging as a process of maintaining and promoting intrinsic capacity instead of merely the absence of illnesses. To better operationalize the intrinsic capacity concept, a novel model of integrated care for older adults (ICOPE) was published by WHO [4]. Central to the ICOPE model is assessing and promoting intrinsic capacity, namely the locomotion, cognition, psychological well-being, vitality, sensory (visual and hearing) capacity, and the social and physical environment an individual engages with to contribute to healthy aging [5].

Improving older adults’ intrinsic capacity has several health implications. It considers capacity change as a continuum through a life course and advocates pursuing optimal functional conditions for all older adults with or without illnesses [6]. It also acknowledges the individuality that people with the same illness condition can experience distinct functional consequences, or people with multi-morbidities require an integrated approach to functional management [7]. Previous studies showed that intrinsic capacity impairment affect self-care, increasing dependence, hospitalization, and mortality among older adults [8, 9]. However, how intrinsic capacity is connected with psychosocial well-being (such as social engagement or loneliness) is unclear. A recent discussion paper reported that lockdown and limited social interactions during the COVID-19 pandemic posed a greater risk to older adults to psychological distress [10]. Research relating to psychosocial well-being in old age is worthy to be supported. Existing studies affirmed biological rather than chronological age on self-care independence; however, the evidence was insufficient beyond the inclusion of individual attributes, psychosocial aspects (i.e., social engagement and loneliness) and intrinsic capacity.

Previous studies [8, 9] provide important insights on the role of intrinsic capacity for older adults; however, more primary studies that adopt ICOPE within different contexts and healthcare systems from screening, full assessment, care planning, and referral are needed. Still, there is less agreement on how the domains of intrinsic capacity should be screened and assessed, and international validation studies are ongoing particularly as Step 2 full assessment is often lacking. The prevalence of intrinsic capacity impairment, to be evaluated by WHO Step 2 full assessment, remains unclear although this assessment is using reliable and validated instruments. Thus, this study aims to assess the level of intrinsic capacity among community-dwelling older adults and examine its relationship with self-care capacity and social engagement. More specifically, the study aimed to answer:

- What is the level of intrinsic capacity impairment in community-dwelling Chinese older adults in Hong Kong?
- What is the sensitivity and specificity of WHO ICOPE Step 1 screening tool in detecting intrinsic capacity impairment when using ICOPE Step 2 full assessment as gold standard?
- What role does intrinsic capacity play in the relationship with self-care capacity, social engagement and demographic factors?

## Methods

### Study design and participants

This study used a cross-sectional design. A convenience sample of community-dwelling older adults were invited to join the study between April to September 2021. Eligible participants were aged over 60 years, living in their own homes, and required no or some support with self-care (feeding, bathing, dressing, toileting). Participants with acute cardiovascular diseases, acute infection, organ dysfunction, dementia, acute mental illness, nearly total-dependent or total-dependent in self-care and being unable to communicate in Cantonese were excluded. The sample size required for Structural Equation Modeling (SEM) depends on the number of factors and the number of indicators [11]. In this study, there were three factors (or called latent variables) and sixteen indicators (or called observed variables). Using the online Free Statistics Calculator version 4 [12] derived from the recommendations for lower bounds on sample size calculation in SEM [13]; and assuming the anticipated effect size of 0.3, desired statistical power level of 0.95, and probability level of 0.05 (type I error), the minimum sample size for this study was 184.

The study was conducted in accordance with the Declaration of Helsinki, and ethical approval was granted from the Human Subjects Ethics Sub-committee of the Hong Kong Polytechnic University (HSEARS20210226005). Written informed consent was obtained from all participants prior to data collection.

### Procedure

A two-day workshop was arranged to 33 nursing student volunteers to train them to conduct a comprehensive assessment and brief care planning based on the ICOPE framework. Theoretical teaching, role play and lab simulation were offered. In the last session of the workshop, these volunteers were evaluated individually on their knowledge and skills mastery. COVID-19 control measures, such as undergoing COVID-19 self-test within 7 days before going to the community centres, social distancing, hand hygiene, wearing masks, and 14-day self-surveillance, were arranged to prevent any spread of COVID-19 to older adults. Regular online meetings and emails were made between the project team and the coordinators of the community centres. The centers’ staff disseminated the study information through phone calls and word of mouth in the community and arranged individual appointments with potential participants. The nursing students explained the study, collected written informed consent, and conducted the nine-item screening, full assessment, and care planning sequentially with the participants. After assessment, the students worked collaboratively with the participants to develop personal goals and brief action plans based on the ICOPE handbook and local support services. A goal-driven action plan booklet was provided to participants as a reminder. For example, participants with cognitive impairment were provided with tips for promoting cognitive health (e.g. reading, exercise, social activities), those with low score in social engagement were assisted to work out brief action plans to engage in favorable activities (e.g., having afternoon tea with family every week). The students referred those older adults who had intrinsic capacity impairment that required further diagnostic assessment to the center staff for follow up and/or further referral as necessary.

To ensure data quality, briefing and debriefing sessions were held for each group of students with 2–3 members before and after their work in the community centres. It took 60 ~ 70 min for each older adult to finish the whole procedure, among which 50 to 60 min was spent on assessment and the rest of the time was spent on the discussion of a brief individual care plan derived from the assessment. The students who enrolled in the project were rewarded with academic credits as part of their clinical placement duties for the community health course.

### Measurements

Participants' demographic and health profiles were collected, including age, gender (males and females), education level, marital status, living conditions, smoking and drinking behavior, and medical history. Participants were also assessed on whether they needed financial support and accommodation support. The participants' medical history was reviewed using the Charlson Comorbidities Index (CCI), which is the summation of the assigned weights of 17 comorbid conditions (myocardial infarction, congestive heart failure, peripheral vascular disease, cerebrovascular disease, dementia, chronic pulmonary disease, connective tissue disease, ulcer disease, mild liver disease and diabetes, hemiplegia, moderate or severe renal disease, diabetes with end organ damage, any malignancy, moderate or severe liver disease (e.g., cirrhosis with ascites), metastatic solid tumor and AIDS) reported by the participants [14]. The CCI is selected for its advantage of reflecting different combinations and severity of diseases [15]. However, as CCI contains no risk factors, we set up an item in the survey to check for the presence of hypertension, the most frequently assessed risk factor in multi-morbidity [16]. The intrinsic capacity was assessed in two steps, including an initial brief screening and a detailed full assessment.

#### Step 1 screening for intrinsic capacity impairment

According to the WHO ICOPE guidelines [4], nine items were used in Step 1 brief screening tool to detect any signs of impairment in six conditions associated with intrinsic capacity: cognitive impairment, limited mobility, malnutrition, visual impairment, hearing loss and depressive symptoms. To minimize the participants’ assessment burden, the same items (such as five-time chair-stand test and whisper test) from the ICOPE screening and the subsequent full assessment were conducted once. Those who had signs of loss in any one of the conditions will proceed to Step 2 full assessment.

#### Step 2 full assessment

##### Cognition: Montreal Cognitive Assessment (MoCA)

The cognitive function was measured by the MoCA, which is translated and validated among Chinese older adults [17]. The MoCA-Chinese version has adequate sensitivity and specificity in differentiating individuals with mild cognitive impairment (≥ 22 and < 26) from those with dementia (< 22, sensitivity: 93.2% and specificity: 71.7%) and normal cognitive function (≥ 26, sensitivity: 92.4% and specificity: 88.4%). One point was added if the person has a primary school education or less, and participants scoring below 22 required further diagnostic assessment.

##### Vitality: Mini Nutritional Assessment (MNA)

The MNA is a 6-item scale which includes anthropometric assessment (weight, height, and weight loss), mobility, dietary intake and health condition, and it is validated in Chinese populations [18]. The MNA score ranges from 0 to 14, with a score of 12 or higher indicating satisfactory nutritional status; a score of eight to 11 indicating a risk of malnutrition; and a score below eight indicating malnutrition in need of further diagnostic assessment.

##### Locomotion

Locomotion capacity was measured by the Short Physical Performance Battery (SPPB), including a hierarchical test of standing balance, a 4-m walk test, and five repetitive chair stands [19]. It has a reliability ranging from 0.88 to 0.92 [20]. Participants’ scoring less than ten indicates limited mobility that requires further diagnostic assessment. Physical activity is measured by the 2-item Mobile Phone Physical Activity Level Questionnaire (MobilePAL), assessing the activity during daytime and nighttime. The MobilePAL is a reliable and valid measure of physical activity that correlated moderately with measurements made by accelerometers (*r* = 0.45; *p* = 0.01) [21]. Participants who reported sitting and standing both during daytime and nighttime were categorized as having a sedentary lifestyle. Grip strength was measured using a hand-held dynamometer; older adults were asked to squeeze the dynamometer with all of their strength three times with each hand. An average score was calculated using the measurements from the dominant hand. The grip strength was adjusted by age and gender, using a cut-off score of the 25^th^ percentile across different age groups and gender among community-dwelling older adults in Hong Kong [22], and were categorized into normal or weak.

##### Psychological well-being: Patient Health Questionnaire (PHQ-9)

The PHQ-9 is a well-validated tool for screening depressive symptoms. PHQ-9 contains nine items on a 4-point Likert scale (marked 0 to 3), with higher scores indicating more severe depression. A score of 10 or above or reported suicidal ideas are considered in need of further diagnostic assessment, and a score between 5 to 9 indicates mild depression [23].

##### Hearing capacity

In addition to the whisper test, the Weber and Rinne test was conducted to detect conductive or sensorineural hearing loss. The participants who correctly repeated the whisper words and passed both the Weber and Rinne test were considered to have intact hearing capacity. Otherwise, further diagnostic assessment was needed.

##### Visual capacity

Visual capacity was measured by WHO simple eye chart considering distance acuity worse than 6/18 as moderate vision impairment that needs further diagnostic assessment. The near vision was assessed by the WHO simple eye chart (near vision) [4]. When the participants’ near visual acuity was worse than N6 or M.08 with correction, impaired visual capacity was noted and further diagnostic assessment was needed [4].

##### Self-care capacity

Self-care was assessed by six items in relation to walking, toileting, dressing, showering, personal appearance, and food preparation. A 3-point Likert scale (1 = independent, 2 = need some support, and 3 = dependent) was used to determine the level of support needed, with a higher score indicating more prominent need of support.

##### Social engagement

Social engagement was assessed by one item asking the extent of pursuing leisure interests, hobbies, work, volunteering, supporting family, educational or spiritual activities that are important to the participant. A 3-point Likert scale was used to determine the level of engagement (1 = inactive, 2 = less active, 3 = active), with a higher score indicating being more active.

##### Loneliness

Loneliness was measured by one item by asking if older adults feel lonely using a 3-point Likert scale (0 = not lonely, 1 = a bit lonely, 2 = very lonely).

#### Data analysis

Descriptive statistics were used to summarize older people's intrinsic capacity and to identify which domains had impairment. Between-group differences were evaluated using the Chi-square test, independent t-test according to the type and normality of the data. Each domain without intrinsic capacity impairment was given one score. That is, participants with MoCA less than 22; MNA less than eight, SPPB less than ten, PHQ-9 above ten or reporting suicidal ideas, distant eye chart worse than 6/18 or a near visual test worse than N6 or M.08 with correction, or failing in any of the hearing tests were scored zero. The intrinsic capacity score ranged from zero to six, with a higher score indicating better intrinsic capacity [24]. Sensitivity and specificity of the ICOPE screening tool in relation to the full assessment was assessed through the area under the curve of the receiver operating characteristic (ROC).

Structural equation modelling was used to determine which variables affect intrinsic capacity. Participants’ characteristics that showed a significant bivariate relationship with intrinsic capacity were entered into the structural equation modelling. Marital status and loneliness were entered to the model initially, but they were excluded as they were not significantly related to intrinsic capacity and the model fit was low. The bivariate association was used as statistical criterion for variable selection of the structural equation modeling (SEM). Chronic illnesses were theoretically connected with intrinsic capacity and therefore hypertension was added to the model. These two methods were used to ensure theoretical and statistical foundation of the model. Age, gender and education were considered as the co-variates. We checked their associations with intrinsic capacity using bivariate analysis and the significant factors were put in the SEM. The model was considered as good fitting when having RMSEA < 0.07, Chi-squared *p*-value > 0.05; Chi-Square/degree of freedom (CMIN/DF) < 3; goodness-of-fit (GFI), comparative fit index (CFI), and Adjusted Goodness of Fit Index (AGFI) > 0.95 [25, 26]. SPSS (Chicago, IL, USA) and SPSS Amos software version 26 were used to conduct the above analysis, with a *p*-value of < 0.05 considered as statistically significant.

## Results

### Demographic characteristics of the sample

The study included 304 older adults and the percentage of participants aged 60 to 69 years, 70 to 79 years, and 80 years or above were 17.1%, 44.4%, and 38.5%, respectively. Most of the participants were females, retired, had education below primary level, and co-resided with family. Less than half (42.2%) of the participants were married and an additional 44.1% were widowed. Majority of the participants (64.1%) reported no pre-defined comorbid conditions in CCI; however, about a quarter had diabetes (*n* = 83, 27.3%) and small number of participants had cardiovascular illness (*n* = 17, 5.6%), malignant tumors (*n* = 15, 4.9%), and liver and kidney diseases (*n* = 11, 3.6%). Hypertension was not included in CCI, it was noted that 132 (43.4%) participants reported having hypertension. Eight participants (2.6%) had psychological distress. Table 1 summarizes the sample characteristics across participants with and without intrinsic capacity impairment.

**Table 1:** Characteristics of participants with or without intrinsic capacity impairment (*n* = 304)

Variables	Without intrinsic capacity impairment	With intrinsic capacity impairment	χ^2^ /t/z	*p*-value
Age	74.52 (6.41)	78.07 (7.29)	5.26	0.02
Gender ##				
Male	17 (29.82%)	40 (70.18%)		
Female	75 (31.65%)	162 (68.35%)	0.07	0.87
Education ##				
Illiterate	3 (5.77%)	49 (94.23%)		
Primary	45 (33.83%)	88 (66.17%)		
Secondary	15 (34.10%)	29 (65.90%)		
High school or above	29 (44.62%)	36 (55.38%)	21.7	< 0.001
Employment ##				
Retried	89 (30.69%)	201 (69.31%)		
Full-time or part-time	3 (75.0%)	1 (25.0%)	3.60	0.09
Marital status ##				
Married	48 (39.34%)	74 (60.66%)		
Single	7 (28.0%)	18 (72.0%)		
Divorced	5 (33.33%)	10 (66.67%)		
Widowed	32 (24.24%)	100 (75.76%)	6.89	0.08
Living status ##				
Living alone	31 (26.72%)	85 (73.28%)		
With family	61 (34.27%)	117 (65.73%)	1.86	0.20
Finance				
Manageable	86 (33.20%)	173 (66.80%)		
Need support	9 (25.0%)	27 (75.0%)	3.50	0.45
Drinking ##				
Yes	5 (33.33%)	10 (66.67%)		
No	87 (31.18%)	192 (68.82%)	0.01	1.00
Smoking ##				
No	92 (31.51%)	200 (68.49%)	1.20	0.53
Body mass index #	22.91 (3.35)	24.13 (4.07)	2.54	0.11
Charlson Comorbidity Index (CCI) #	0.55 (0.91)	0.49 (0.82)	0.58	0.45
Hypertension ##				
Yes	33 (25.58%)	96 (74.42%)		
No	59 (35.76%)	106 (64.24%)	3.49	0.08

Note. # t-test, ##chi-square test were used

### Intrinsic capacity of the participants

#### Step 1 WHO ICOPE screening

Using the ICOPE screening tool, 221 participants (72.7%) were found to have impairment in any domain in intrinsic capacity. The percentage of participants having impaired locomotion, cognition, psychological well-being, vitality, sensory capacity (visual) and sensory capacity (hearing capacity) was 37.8%, 24.3%, 35.2%, 18.1%, 8.9% and 14.5% respectively (Table 2).

**Table 2:** Intrinsic capacity impairment in WHO ICOPE Step 1 screening (*n *= 304) and Step 2 full assessment (*n *= 296)

Variables	Step 1 screening	Step 2 full assessment
Intrinsic capacity impairment	72.7% (*n* = 221)	66.4% (*n* = 202)
***Domains of intrinsic capacity***		
Locomotion	37.8% (*n* = 115)	39.8% (*n* = 117)
Cognition	24.3% (*n* = 74)	25.5% (*n* = 75)
Psychological well-being	35.2% (*n* = 107)	11.6% (*n* = 34)
Vitality	18.1% (*n* = 55)	2.7% (*n* = 8)
Sensory capacity (Vision)	8.9% (*n* = 27)	24.7% (*n* = 75)
Sensory capacity (Hearing)	14.5% (*n* = 44)	27.9% (*n* = 82)

#### Step 2 full assessment

A total of 304 participants attended the full assessment with an average intrinsic capacity score of 4.68 (SD = 1.25), and 202 participants (66.4%) were categorized as having one or more impaired intrinsic capacity domains. The percentage of impairment in locomotion, cognition, psychological well-being, vitality, sensory capacity (vision), and sensory capacity (hearing) was 39.8% (*n* = 117), 25.5% (*n* = 75), 11.6% (*n* = 34), 2.7% (*n* = 8), 24.7% (*n* = 75), and 27.9% (*n* = 82), respectively. Of the participants, 86 (28.3%), 63 (20.7%), 32 (10.5%), 13 (4.3%), and 6 (2.0%) showed an impairment in one, two, three, four, and five domains, respectively. A total of 50 (17%) participants had both impairment in locomotion and cognition. Another 16.6% (*n* = 49) of the participants had mild cognitive impairment, were at risk of malnutrition, or had mild depression.

**Fig. 1:**
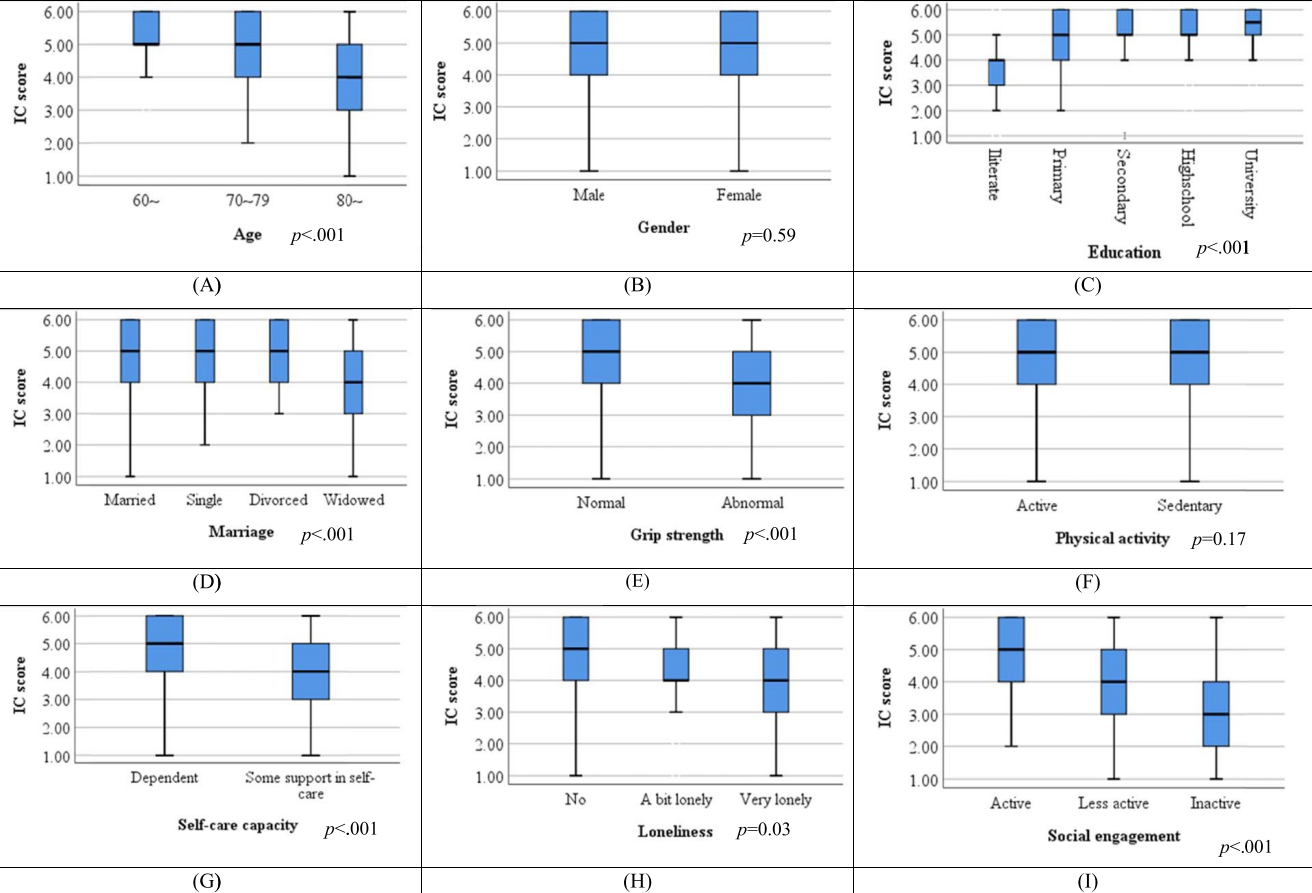
Comparison of intrinsic capacity (IC) scores among different groups. **A** age groups, (**B**) gender, (**C**) education, (**D**) marital status, (**E**) grip strength, (**F**) physical activity (**G**) self-care capacity, (**H**) loneliness and (**I**) social engagement

Older participants showed lower intrinsic capacity on average, and the mean score ranged from 5.30 (SD = 0.75) (at age 60 to 69) to 4.07 (SD = 1.39) (at age 80 and older, *p* < 0.001) (Fig. 1A). There was no significant difference in intrinsic capacity between males and females (Fig. 1B). The education level (*p* < 0.001) and marital status (*p* < 0.001) were both significantly associated with intrinsic capacity score (Fig. 1C, D), but not living status. Participants with weak grip strength (*p* < 0.001) had significantly lower intrinsic capacity, but the difference was not significant in the physical activity level (Fig. 1E, F). Participants who needed some support in self-care in activities of daily living (*p* < 0.001), felt a bit or very lonely (*p* = 0.003), or were less active in social engagement (*p* < 0.001), had significantly lower intrinsic capacity (Fig. 1G-I).

### Sensitivity and specificity of the WHO ICOPE screening tool

Table 3 shows the contingency table for the positive full assessment results and the screening results. The sensitivity and specificity of the ICOPE screening tool to detect one or more intrinsic capacity impairment were 95% and 57.6%. Furthermore, the sensitivity of all domains ranged from 74.7% in cognition to 100% in vitality and hearing. For the 19 older adults who showed no signs of cognitive impairment in the brief screening but scored below 22 in MoCA, 11 were illiterate, 7 had primary school education, and all lost scores on visual and executive items of the MoCA. The specificity of the overall ICOPE screening tool was relatively low (57.6%) and specificity of individual domains (ranging from 74% to 97.4%) of the ICOPE screening was good.

**Table 3:** Sensitivity and specificity of intrinsic capacity and its five domains

Overall intrinsic capacity		Full assessment			
ICOPE screening		+	-	Total	
	+	192	39	231	Sensitivity = 95.0%
	-	10	53	63	Specificity = 57.6%
	Total	202	92	294	
Cognitive capacity					
ICOPE screening		MoCA			
		+	-		
	+	56	18	74	Sensitivity = 74.7%
	-	19	211	230	Specificity = 92.1%
	Total	75	229	304	
Locomotion capacity					
ICOPE screening		SPPB			
		+	-		
	+	102	13	115	Sensitivity = 85%
	-	18	167	185	Specificity = 92.8%
	Total	120	180	300	
Vitality capacity					
ICOPE screening		MNA			
		+	-		
	+	8	47	55	Sensitivity = 100%
	-	0	249	249	Specificity = 84.1%
	Total	8	296	304	
Psychological capacity					
ICOPE screening		PHQ-9			
		+	-		
	+	186	76	262	Sensitivity = 98.4%
	-	3	31	34	Specificity = 74.0%
	Total	189	107	296	
Sensory capacity (Vision)					
ICOPE screening		Distal & proximal visual test			
		+	-		
	+	25	46	71	Sensitivity = 86.2%
	-	4	223	227	Specificity = 82.9%
	Total	29	269	300	
Sensory capacity (Hearing)					
ICOPE screening		Whisper, Weber & Rinne test			
		+	-		
	+	44	38	82	Sensitivity = 100%
	-	0	222	222	Specificity = 85.4%
	Total	44	260	304	

The ROC curve analysis was performed to investigate the performance of the ICOPE brief screening tool in relation to the dichotomized (one or more impaired domains versus no impairment) full-assessment results. Using a cutoff point of 5 (i.e. at least one domain with intrinsic capacity impairment), the sensitivity and specificity of the ICOPE screening tool in identifying older adults with the intrinsic capacity impairment were 90% and 42%, respectively. The ROC area for the ICOPE screening tool was 0.853 (*p* < 0.001).

### Intrinsic capacity and its relationship with other study variables

**Fig. 2:**
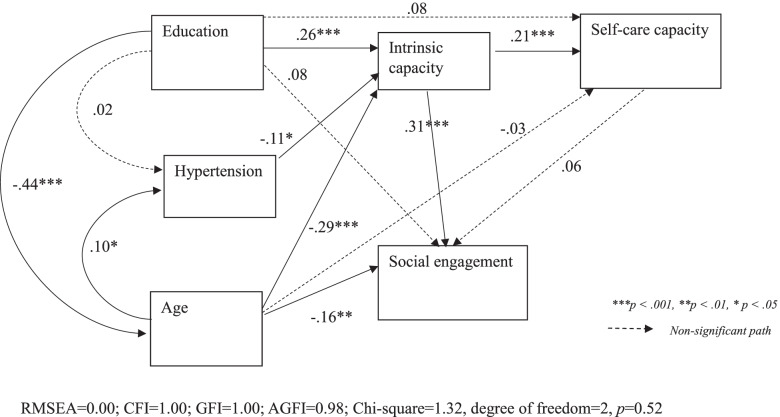
Model showing the relationships among intrinsic capacity, social engagement and self-care capacity

The structural equation modelling showed a good fitness, with fit indices: RMSEA = 0.00; CFI = 1.00; GFI = 1.00; AGFI = 0.98; Chi-square = 1.32, degree of freedom = 2, *p* = 0.52. The final model showed people in younger old age (β = -0.29, *p* < 0.001), with higher education (β = 0.26, *p* < 0.001), with absence of hypertension (β = -0.11, *p* < 0.05) were more likely to have better intrinsic capacity (Fig. 2). The model also showed a significant positive relationship between intrinsic capacity and self-care capacity in performing activities of daily living (β = 0.21, *p* < 0.001). Another positive relationship between intrinsic capacity and social engagement (β = 0.31, *p* < 0.001) was noted. The relationship between self-care capacity and social engagement was not statistically significant. Intrinsic capacity mediates the relationship between education and self-care capacity. Such mediating role also happened in the relationship between age and social engagement.

## Discussion

This study shows the first adaptation of the ICOPE model (including brief screening, full assessment, and care planning) in a convenience sampling in Hong Kong and this indicated the urgency of community-level support to older adults for the high prevalence of intrinsic capacity impairment. The sensitivity and specificity of the ICOPE step 1 screening tool suggested its ability to correctly identify people with one or more impaired intrinsic capacity. Special attention should be paid to the older adults in advanced age, with less education, or those having hypertension because they were more likely to have a greater number of impaired intrinsic capacity domains. In addition, an increased number of impaired intrinsic capacity domains was associated with dependency in activities of daily living and decreased social engagement.

Consistent with previous studies that this study showed a high prevalence of intrinsic capacity impairment among older adults. In France, one WHO collaborating center has conducted a preliminary screening of intrinsic capacity using a nine-item ICOPE screening tool. Results indicated that 699 (92.6%) of 755 older people had intrinsic capacity impairment in at least one domain that required full assessment [27]. Another study in China assessed 376 older adults using the same nine items, and 69.1% of older adults had impairment in at least one intrinsic capacity domain [24]. A secondary analysis using China Health and Retirement Longitudinal Study (CHARLS) dataset showed a significant negative relationship between intrinsic capacity and self-care in activities of daily living and instrumental activities of daily living [28]. All these findings indicated the importance of the attention to intrinsic capacity in old age and the pressing need for care pathway establishment for further referral.

It is encouraging that the step 1 ICOPE screening can correctly identifying intrinsic capacity impairment, which permits assessors to be confident to recommend older adults to be referred and undergo full assessment when the screening tests yield positive results. The nine-item ICOPE screening rendered it an ideal tool for manpower-saved assessment, implying wide applicability for community-level intrinsic capacity assessment. Literature also supported using the step 1 ICOPE screening tool for intrinsic capacity assessment as it was correlated with physical, mental and organ functions among older adults [24] as well as with physicians’ clinical judgement [29]. Considering the relatively lower sensitivity of the cognition screening items, adding visual and executive items may further enhance its sensitivity. Using a cutoff score of five means that one or more impaired domains detected by the step 1 screening may imply one or more impaired intrinsic capacity domains evaluated by the full assessment. However, the cutoff score generated in this study is different from a previous study that recommended using two or more domains with signs of impairment in relation to performance in activities of daily living and instrumental activities of daily living [24]. Findings from the current study suggested that any domain with signs of intrinsic capacity impairment should arouse attention 1) to prevent deconditioning [4, 5], and 2) considering the uniqueness of each domain for older adults to resume self-care and maintain independence [28].

The findings affirmed literature’s emphasis on the negative consequences of intrinsic capacity impairment on self-care insufficiency among older adults, and added empirical evidence of its impairment towards social engagement, which rendered intrinsic capacity as a biopsychosocial construct [30]. One study analyzed data from the Health and Retirement Study (*n* = 11,093) and demonstrated that cognition, vitality, vision, and hearing impairment were associated with dependency on activities of daily living [31]. Another longitudinal study reported that impairments in locomotion, cognitive, visual, hearing, and psychological capacity were associated with an increased risk of mobility disability (i.e., difficulty/unable to climb ten steps or walk 1/2 mile) or dependency on activities of daily living [32]. A systematic review identified domains of intrinsic capacity as protective factors for independence in activities of daily living among older adults [33]. Recent studies that operationalized the intrinsic capacity construct with all domains consistently highlighted the contribution to the independence of activities of daily living through path analysis [24, 28].

Lower number of impaired intrinsic capacity domain was positively related to social engagement, which is crucial for older adults to live an active lifestyle and gain health benefits [34]. Literature has individually examined the negative influence of impairment in every five domains on social engagement; for example, hearing loss might lead to social isolation, mobility problems may prohibit social activities engagement (i.e., group dance) [35]. However, findings from this study suggest that five domains of intrinsic capacity should not be regarded as standalone silos in influencing social engagement. Each domain closely interacts with others as a dynamically interrelated environment that the reciprocal and synergistic effects of impairment in intrinsic capacity should be evaluated in enhancing social engagement among older adults [36].

The findings of the structural equation model also suggest intrinsic capacity is a mediator in the relationship between education and self-care capacity and the relationship between age and social engagement. People with high education level do not necessarily possess good self-care capacity; however, through the possession of intrinsic capacity, people with different education levels were capable to perform self-care in old age. This finding is important because interventions should be developed to build up older adults’ intrinsic capacity regardless of their educational levels. Similarly, as intrinsic capacity was found to be the mediator of the relationship between age and social engagement, we should develop programs to build up older adults’ intrinsic capacity (even though they are at advanced age). A few interventions [37, 38] have been developed to enhance intrinsic capacity in old age. Further investigation and development would be needed to show the efficacy of these interventions.

To prevent or slow down intrinsic capacity deterioration and its consequence on self-care insufficiency and social disengagement, it is crucial to assess intrinsic capacity holistically [39]. Step 1 screening tool, with its good sensitivity and easy administration, can be an ideal kick-start to capture intrinsic capacity conditions of older adults, and this can be carried out by the people who are not professionals but well-trained. Step 1 brief screening can be easily conducted by trained volunteers who may mitigate the manpower shortage in aging services [29]. However, brief screening alone cannot specify the support older adults need and are entitled to. It is necessary to provide a subsequent full assessment if the screening shows the sign of impairment in any domain of intrinsic capacity. Such assessment can determine whether older adults self-manage (or manage with guidance) their daily lives at home or require further supports from professionals in diagnosis and treatment.

### Implications

This study has several key practice and research implications. A high proportion of community-dwelling older adults were living with actual or were at risk of intrinsic capacity impairment, but no assessment has been carried out regularly with them. There is a high demand for ICOPE trained personnel that 72.7% of older adults were detected with impairment in at least one intrinsic capacity domain, and two-thirds needed an in-depth assessment. The WHO ICOPE brief screening tool allows for quick assessment that can be conducted by non-healthcare professionals such as trained volunteers to identify those in need of in-depth assessment and/or further referral. Moreover, once the impairments are detected, the health care system should provide intervention using an inter-disciplinary healthcare team to slow progression and prevent subsequent limitations on self-care capacity in performing activities of daily living. Integrating the ICOPE model at community level in Hong Kong can be challenging; thus, engaging stakeholders (policymakers, managers, and health care professionals) is crucial for the success of the ICOPE care pathway establishment. In addition, older adults and family members should be informed of these impairments with the education provided to enhance self-management skills and foster common recognition of the concept of healthy aging.

### Limitations

The study used a convenience sampling method which may impair the generalizability of the results. Although we are not using a random sampling, we have already tried the best to conduct a multi-site data collection (collecting data from 11 community centres out of 41 centres in Hong Kong). To some extent, this is the best sample we can obtain in the region. It is challenging to recruit a random sample during the COVID-19 pandemic. The decreased willingness of older adults to participate in a research study with face-to-face interactions during COVID-19 may also compromise sample representativeness [40]. Other limitations may arise from the design of this study in which assessors conducted the brief screening and full assessment for all the participants, which prolonged the duration of the assessment. The time devoted to the discussion with the older adults about the care plan was short. We could only provide a brief care plan to the older adults in this study. Further studies should focus on the development of person-centred care detailed plans and goal setting sessions so that older adults could express their concerns and the resources that possess to improve their health alongside evaluations of the outcomes of such care planning.

## Conclusions

The high proportion of older adults with intrinsic capacity impairment and the significant contribution to independence in self-care and social disengagement urges the ICOPE intrinsic capacity assessment at the community level as a means to promote early detection of and interventions on intrinsic capacity impairment. The ICOPE screening can be a reliable tool to detect signs of intrinsic capacity, which is promising for large-scale usage. Subsequent comprehensive assessment should be conducted among those with indications intrinsic capacity impairment to determine whether the person can manage them at home or need a referral to professional care. A care pathway involving professionals and family members is needed to offer diagnostic assessment and guide self-management of intrinsic capacity impairment among older adults.

## Data Availability

The datasets used and/or analyzed during the current study are available from the corresponding author on reasonable request.

## Abbreviations

WHO: World Health Organization
ICOPE: Integrated Care for Older People
CCI: Charlson Comorbidities Index
MoCA: Montreal cognitive assessment
MNA: Mini Nutritional Assessment
SPPB: Short Physical Performance Battery
MobilePAL: Mobile Phone Physical Activity Level Questionnaire
PHQ-9: Patient Health Questionnaire
ROC: Receiver operating characteristic
GFI: Goodness-of-fit
CFI: Comparative fit index
AGFI: Adjusted Goodness of Fit Index
